# Exploring Biomolecular Literature with EVEX: Connecting Genes through Events, Homology, and Indirect Associations

**DOI:** 10.1155/2012/582765

**Published:** 2012-06-06

**Authors:** Sofie Van Landeghem, Kai Hakala, Samuel Rönnqvist, Tapio Salakoski, Yves Van de Peer, Filip Ginter

**Affiliations:** ^1^Department of Plant Systems Biology, VIB, Technologiepark 927, 9052 Gent, Belgium; ^2^Department of Plant Biotechnology and Bioinformatics, Ghent University, Technologiepark 927, 9052 Gent, Belgium; ^3^Department of Information Technology, University of Turku, Joukahaisenkatu 3-5, 20520 Turku, Finland; ^4^Turku BioNLP Group, Turku Centre for Computer Science (TUCS), Joukahaisenkatu 3-5, 20520 Turku, Finland

## Abstract

Technological advancements in the field of genetics have led not only to an abundance of experimental data, but also caused an exponential increase of the number of published biomolecular studies. Text mining is widely accepted as a promising technique to help researchers in the life sciences deal with the amount of available literature. This paper presents a freely available web application built on top of 21.3 million detailed biomolecular events extracted from all PubMed abstracts. These text mining results were generated by a state-of-the-art event extraction system and enriched with gene family associations and abstract generalizations, accounting for lexical variants and synonymy. The EVEX resource locates relevant literature on phosphorylation, regulation targets, binding partners, and several other biomolecular events and assigns confidence values to these events. The search function accepts official gene/protein symbols as well as common names from all species. Finally, the web application is a powerful tool for generating homology-based hypotheses as well as novel, indirect associations between genes and proteins such as coregulators.

## 1. Introduction

The field of natural language processing for biomolecular texts (BioNLP) aims at large-scale text mining in support of life science research. Its primary motivation is the enormous amount of available scientific literature, which makes it essentially impossible to rapidly gain an overview of prior research results other than in a very narrow domain of interest. Among the typical use cases for BioNLP applications are support for database curation, linking experimental data with relevant literature, content visualization, and hypothesis generation—all of these tasks require processing and summarizing large amounts of individual research articles. Among the most heavily studied tasks in BioNLP is the extraction of information about known associations between biomolecular entities, primarily genes, and gene products, and this task has recently seen much progress in two general directions.

First, relationships between biomolecular entities are now being extracted in much greater detail. Until recently, the focus was on extracting untyped and undirected binary relations which, while stating that there is *some* relationship between two objects, gave little additional information about the nature of the relationship. Recognizing that extracting such relations may not provide sufficient detail for wider adoption of text mining in the biomedical community, the focus is currently shifting towards a more detailed analysis of the text, providing additional vital information about the detected relationships. Such information includes the type of the relationship, the specific roles of the arguments (e.g., affector or affectee), the polarity of the relationship (positive versus negative statement), and whether it was stated in a speculative or affirmative context. This more detailed text mining target was formalized as an *event extraction* task and greatly popularized in the series of BioNLP Shared Tasks on Event Extraction [1, 2]. These shared tasks mark a truly community-wide effort to develop efficient systems to extract sufficiently detailed information for real-world, practical applications, with the highest possible accuracy.

Second, text mining systems are now being applied on a large scale, recognizing the fact that, in order for a text mining service to be adopted by its target audience, that is, researchers in the life sciences, it must cover as much of the available literature as possible. While small-scale studies on well-defined and carefully constructed corpora comprising several hundred abstracts are of great utility to BioNLP research, actual applications of the resulting methods require the processing of considerably larger volumes of text, ideally including all available literature. Numerous studies have been published demonstrating that even complex and computationally intensive methods can be successfully applied on a large scale, typically processing all available abstracts in PubMed and/or all full-text articles in the open-access section of PubMed Central. For instance, the *iHOP* [3] and *Medie* [4] systems allow users to directly mine literature relevant to given genes or proteins of interest, allowing for structured queries far beyond the usual keyword search. *EBIMed* [5] offers a broad scope by also including gene ontology terms such as biological processes, as well as drugs and species names. Other systems, such as the *BioText search engine* [6] and *Yale Image Finder* [7] allow for a comprehensive search in full-text articles, including also figures and tables. Finally, the *BioNOT* system [8] focuses specifically on extracting negative evidence from scientific articles.

The first large-scale application that specifically targets the extraction of detailed events according to their definition in the BioNLP Shared Tasks is the dataset of Björne et al. [9], comprising 19 million events among 36 million gene and protein mentions. This data was obtained by processing all 18 million titles and abstracts in the 2009 PubMed distribution using the winning system of the BioNLP'09 Shared Task. In a subsequent study of Van Landeghem et al. [10], the dataset was refined, generalized, and released as a relational (SQL) database referred to as *EVEX*. Among the main contributions of this subsequent study was the generalization of the events, using publicly available gene family definitions. Although a major step forward from the original text-bound events produced by the event extraction system, the main audience for the EVEX database was still the BioNLP community. Consequently, the dataset is not easily accessible for researchers in the life sciences who are not familiar with the intricacies of the event representation. Further, as the massive relational database contains millions of events, manual querying is not an acceptable way to access the data for daily use in life science research.

In this study, we introduce a publicly available web application based on the EVEX dataset, presenting the first application that brings large-scale event-based text mining results to a broad audience of end-users including biologists, geneticists, and other researchers in the life sciences. The web application is available at http://www.evexdb.org/. The primary purpose of the application is to provide the EVEX dataset with an intuitive interface that does not presuppose familiarity with the underlying event representation. The application presents a comprehensive and thoroughly interlinked overview of all events for a given gene or protein, or a gene/protein pair. The main novel feature of this application, as compared to other available large-scale text mining applications, is that it covers highly detailed event structures that are enriched with homology-based information and additionally extracts indirect associations by applying cross-document aggregation and combination of events.

In the following section, we provide more details on the EVEX text mining dataset, its text-bound extraction results, and the gene family-based generalizations. Further, we present several novel algorithms for event ranking, event refinement, and retrieval of indirect associations.  Section 3 presents an evaluation of the EVEX dataset and the described algorithms. The features of the web application are illustrated in Section 4, presenting a real-world use case on the budding yeast gene *Mec1*, which has known mammalian and plant homologs. We conclude by summarizing the main contributions of this work and highlighting several interesting opportunities for future work.

## 2. Data and Methods

This section describes the original event data, as well as a ranking procedure that sorts events according to their reliability. Further, two abstract layers are defined on top of the complex event structures, enabling coarse grouping of similar events, and providing an intuitive pairwise point of view that allows fast retrieval of interesting gene/protein pairs. Finally, we describe a hypothesis generation module that finds missing links between two entities, allowing the user to retrieve proteins with common binding partners or genes that act as coregulators of a group of common target genes.

### 2.1. EVEX Dataset

#### 2.1.1. Core Events

The core set of text mining results accessible through the EVEX resource has been generated by the Turku Event Extraction System, the winning system of the BioNLP'09 Shared Task (ST) on Event Extraction [1]. This extraction system was combined with the BANNER named entity recognizer [11], forming a complete event extraction pipeline that had the highest reported accuracy on the task in 2009, and still remains state-of-the-art, as shown in the recent ST'11 [12]. This event extraction pipeline was applied to all citations in the 2009 distribution of PubMed [9]. As part of the current study, citations from the period 2009–2011 have been processed, using essentially the same pipeline with several minor improvements, resulting in 40.3 million tagged gene symbols and 21.3 million extracted events. The underlying event dataset has thus been brought up to date and will be regularly updated in the future.

**Figure 1:**
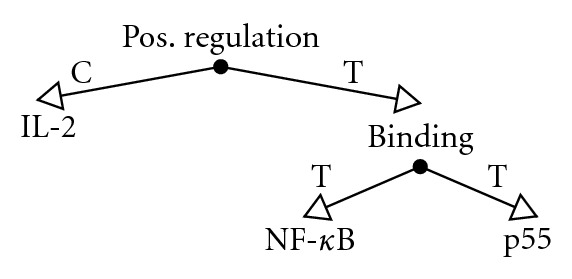
Event representation of the statement *IL-2 acts by enhancing binding activity of NF-κB to p55*, illustrating recursive nesting of events where the (T)heme of the *positive regulation* event is the *binding* event. The (C)ause argument is the gene symbol *IL-2* (figure adapted from [10]).

The dataset contains events as defined in the context of the ST'09, that is, predicates with a variable number of arguments which can be gene/protein symbols or, recursively, other events. Each argument is defined as having the role of *Cause* or *Theme* in the event. There are nine distinct event types: binding, phosphorylation, regulation (positive, negative, and unspecified), protein catabolism, transcription, localization, and gene expression. Further, each event refers to a specific *trigger word* in text. For example, the word *increases* typically triggers a positive regulation event and *degradation* typically refers to protein catabolism. An example event structure is illustrated in Figure 1.

Event definitions impose several restrictions on event arguments: (1) events of the type phosphorylation, protein catabolism, transcription, localization, and gene expression must only have a single argument, a Theme, which must be a gene or a protein, (2) events of the binding type may have any number of gene/protein Theme arguments and cannot have a Cause argument, and finally (3) regulation events must have exactly one Theme argument and may have one Cause argument, with no restrictions as to whether these arguments are genes/proteins or recursively other events. In the following text, we will state events using a simple bracketed notation, where the event type is stated first, followed by a comma-separated list of arguments enclosed in parentheses. For instance, the event in Figure 1 would be stated as *Positive-Regulation(C:IL-2, T:Binding(T:NF-κB, T:p55))*, where *C:* and *T:* denote the role of the argument as (C)ause or (T)heme. For brevity, we will further refer to all biochemical entities, even proteins and mRNA, as *genes*.

#### 2.1.2. Event Generalizations

 One of the major limitations of the original core set of events is that they are strictly text-bound and provide no facility for a more general treatment, such as being able to abstract from different name spelling variants and symbol synonymy. Further, biochemical entities were originally treated as merely text strings with no database identity referring to external resources such as UniProt [13] or Entrez Gene [14]. The EVEX dataset addresses these issues by providing event generalizations [10].

First, the identified gene symbols in the EVEX dataset are canonicalized by removing superfluous affixes (prefixes and suffixes) to obtain the core gene symbol, followed by discarding nonalphanumeric characters and lowercasing. For instance, the full string *human Esr-1 subunit* is canonicalized into *esr1*. The purpose of this canonicalization is to abstract away from minor spelling variants and to deal with the fact that the BANNER named entity recognizer often includes a wider context around the core gene symbol. The canonicalization algorithm itself cannot, however, deal with the ambiguity prevalent among the symbols. EVEX thus further resolves these canonical gene symbols, whenever possible, into their most likely families, using two distinct resources for defining homologous genes and gene families: *HomoloGene* (eukaryots, [14]) and *Ensembl* (vertebrates, [15]). As part of this study, we extended EVEX to also include families from *Ensembl Genomes*, which provides coverage for metazoa, plants, protists, fungi, and bacteria [16]. Building on top of these definitions, the EVEX dataset now defines four *event generalizations*, whereby all events whose arguments have the same canonical form, or resolve to the same gene family, are aggregated. As a result, it becomes straightforward to retrieve all information on a specific gene symbol, abstracting away from lexical variants through the canonicalization algorithm, or to additionally apply the synonym-expansion module through the family-based generalizations. These different generalizations are all implemented on the web application (Section 4.2).

### 2.2. Event Ranking

 To rank the extracted events according to their reliability, we have implemented an event scoring algorithm based on the output of the Turku Event Extraction System. This machine learning system uses linear Support Vector Machines (SVMs) as the underlying classifier [17]. Every classification is given a confidence score, the distance to the decision hyperplane of the linear classifier, where higher scores are associated with more confident decisions. There is not a single master classifier to predict the events in their entirety. Rather, individual classifications are made to predict the event trigger and each of its arguments. In order to assign a single confidence score to a specific event occurrence, the predictions from these two separate classifiers must be aggregated.

The confidence scores of the two different classifiers are not directly mutually comparable, and we therefore first normalize all scores in the dataset to zero mean and unit standard deviation, separately for triggers and arguments. Subsequently, the score of a specific event occurrence is assigned to be the *minimum* of the normalized scores of its event trigger and its arguments, that is, the lowest normalized confidence among all classification decisions involved in extracting that specific event. Using minimum as the aggregation function roughly corresponds to the *fuzzy and* operator in that it requires all components of an event to be confident for it to be ranked high. Finally, the score of a generalized event is the average of the scores of all its occurrences.

To assign a meaningful interpretation to the normalized and aggregated confidence values, events within the top 20% of the confidence range are classified as “very high confidence.” The other 4 categories, each representing the next 20% of all events, are respectively labeled as “high confidence,” “average confidence,” “low confidence” and “very low confidence.” When presenting multiple possible hits for a certain query, the web application uses the original scores to rank the events from high to low reliability.

### 2.3. Event Refinement

 The extraction of event structures is highly dependent on the lexical and syntactic constructs used in the sentence and may therefore contain unnecessary complexity. This is because the event extraction system is trained to closely follow the actual statements in the sentence and thus, for instance, will mark both of the words *increase* and *induces* as triggers for positive regulation events in the sentence *Ang II induces a rapid increase in MAPK activity*. Consequently, the final event structure is extracted as *Positive-Regulation(C: Ang II, T: Positive-Regulation(T: MAPK))*, that is, *Ang II* is a Cause argument of a positive regulation event, which has another positive regulation event as its Theme.

While correctly extracted, such nested single-argument regulatory events (i.e., regulations with a Theme but no Cause argument), often forming chains that are several events long, are unnecessarily complex. Clearly, the event above can be restated as *Positive-Regulation(C: Ang II, T: MAPK)*, removing the nested single-argument positive regulation event. This refinement helps to establish the event as equivalent with all other events that can be refined to the same elementary structure, enhancing the event aggregation possibilities in EVEX. However, when presenting the details of the extracted event to the user, the original structure of the event is preserved.

Table 1 lists the set of refinement rules. In this context, positive and negative regulation refer to having a general positive or negative effect, while an unspecified regulation could not be resolved to either category due to missing information in the sentence.

To simplify the single-argument regulatory events, we proceed iteratively, removing intermediary single-argument regulatory events as long as any rule matches. A particular consideration is given to the polarity of the regulations. While a nested chain of single-argument positive regulations can be safely reduced to a single positive regulation, the outcome of reducing chains of single-argument regulations of mixed polarity is less obvious. As illustrated in Table 1, application of the rules may result in a change of polarity of the outer event. For instance, a regulation of a negative regulation is interpreted as a negative regulation, changing the polarity of the outer event from unspecified to negative. To avoid excessive inferences not licensed by the text, the algorithm only allows one such change of polarity. Any subsequent removal of a nested single-argument regulatory event that results in a type change forces the new type of the outer event to be of the unspecified regulation type.

### 2.4. Pairwise Abstraction

The most basic query issued on the EVEX web application involves a single gene, which triggers the generation of a structured overview page, listing associated genes grouped by their type of connection with the query gene (Section 4.1). The most important underlying functionality implemented by the web application is thus the ability to identify and categorize pairs of related genes. This pairwise point of view comes natural in the life sciences and can be implemented on top of the events with ease by analyzing common event structures and defining argument pairs within. The refinements discussed in Section 2.3 substantially decrease the number of unique event structures present in the data, restricting the required analysis to a comparatively small number of event structures. Furthermore, we only need to consider those events that involve more than one gene or that are a recursive argument in such an event, limiting the set of event occurrences from 21 M to 12 M events.

As an example, let us consider the event *Positive-Regulation(C:Thrombin, T:Positive-Regulatio(C:EGF, Phosphorylation(T:Akt)))*, extracted from the sentence *Thrombin augmented EGF-stimulated Akt phosphorylation*. The pairs of interest here are *Thrombin—Akt* and *EGF—Akt*, both associations coarsely categorized as *regulation*. Therefore, whenever a user queries for *Thrombin*, the *Akt* gene will be listed among the regulation targets, and, whenever a user queries for *Akt*, both *Thrombin* and *EGF* will be listed as regulators. Note, however, that the categorization of the association as *regulation* is only for the purpose of coarse grouping of the results on the overview page. The user will additionally be presented with the details of the original event, which is translated from the bracketed notation into the English statement *Upregulation of AKT phosphorylation by EGF is upregulated by Thrombin*.

There is a limited number of prevalent event structures which account for the vast majority of event occurrences. Table 2 lists the most common structures, together with the gene pairs extracted from them. The algorithm to extract the gene pairs from the event structures proceeds as follows.

1. All argument pairs are considered a candidate and classified as *binding* if both participants are a Theme of one specific binding event, and *regulation* otherwise. (Note that due to the restrictions of event arguments as described in Section 2.1, only binding and regulation events can have more than one argument.)
2. If one of the genes is a Theme argument of an event which itself is a Cause argument, for example, *G2* in *Regulation(C:Regulation(C:G1, T:G2), T:G3)*, the association type of the candidate pair *G2-G3* is reclassified as *indirect regulation*, since the direct regulator of *G3* is the Cause argument of the nested regulation (*G1*).
3. If one of the genes is a Cause argument of an event which itself is a Theme argument, for example, *G2* in *Regulation(C:G1, T:Regulation(C:G2, T:G3))*, the candidate pair (*G1-G2*) is discarded.

While the association between *G1* and *G2* is discarded in step (3) since it in many cases cannot convincingly be classified as a regulation, it is represented as a *coregulation* when indirect associations, described in the following section, are sought.

### 2.5. Indirect Associations

 A cell's activity is often organized into regulatory modules, that is, sets of coregulated genes that share a common function. Such modules can be found by automated analysis and clustering of genome-wide expression profiles [18]. Individual events, as defined by the BioNLP Shared Tasks, do not explicitly express such associations. However, indirect regulatory associations can be identified by combining the information expressed in various events retrieved across different articles. For instance, the events *Regulation(C:geneA, T:geneZ)* and *Regulation(C:geneB, T:geneZ)* can be aggregated to present the hypothesis that *geneA* and *geneB* coregulate *geneZ*. Such hypothesis generation is greatly simplified by the fact that the events have been refined using the procedure described in Section 2.3 and the usage of a relational database, which allows efficient querying across events.

The indirect associations as implemented for the web application include coregulation and common binding partners (Table 3). These links have been precalculated and stored in the database, enabling fast retrieval of, for example, coregulators or genes that are targeted by a common regulator, facilitating the discovery of functional modules through text mining information. However, it needs to be stated that these associations are mainly hypothetical, as, for example, coregulators additionally require coexpression. Details on gene expression events can be found by browsing the sentences of specific genes as described in Section 4.1.

## 3. Results and Performance Evaluation

In this section, we present the evaluation of the EVEX resource from several points of view. First, we discuss the performance of the event extraction system used to produce the core set of events in EVEX, reviewing a number of published evaluations both within the BioNLP Shared Task and in other domains. Second, we present several evaluations of the methods and data employed specifically in the EVEX resource in addition to the core event predictions: we review existing results as well as present new evaluations of the confidence scores and their correlation with event precision, the family-based generalization algorithms, and the novel event refinement algorithms introduced above. Finally, we discuss two biologically motivated applications of EVEX, demonstrating the usability of EVEX in real-world use cases.

### 3.1. Core Event Predictions

The Turku Event Extraction System (TEES), the source of the core set of EVEX events, was extensively evaluated on the BioNLP Shared Tasks. It was the winning system of the ST'09, achieving 46.73% recall, 58.48% precision, and 51.95% *F*-score [9]. In the current study, the original set of event predictions extracted from the PubMed 2009 distribution has been brought up to date using an improved version of TEES. This updated system was recently shown to achieve state-of-the-art results in the ST'11, obtaining 50.06% recall, 59.48% precision, and 54.37% *F*-score on the corresponding abstract-only GENIA subchallenge [12].

To assess the generalizability of the text mining results from domain-specific datasets to the whole of PubMed, a precision rate of 64% was previously obtained by manual evaluation of 100 random events [19]. In the same study, the named entities (i.e., gene and protein symbols) as extracted by BANNER were estimated to achieve a precision of 87%. These figures indicate that the performance of the various text mining components generalize well from domain-specific training data to the entire PubMed.

### 3.2. Confidence Values

To investigate the correlation of the confidence values (Section 2.2) to the correctness of the extracted events, we have measured the precision and recall rates of binding events between two genes, simulating a use case that involves finding related binding partners for a certain query gene (Section 4.1). This experiment was conducted on the ST'09 development set, consisting of 150 PubMed abstracts with 94 gold-standard binding pairs. For this dataset, 67 interacting pairs were found in EVEX, with confidence values ranging between −1.7 and 1.3. When evaluated against the gold-standard data, the whole set of predictions achieves 59.7% precision and 42.6% recall.

**Figure 2:**
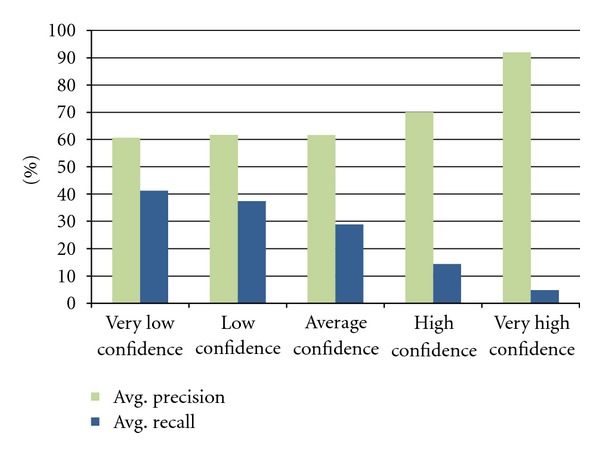
Evaluation of predicted binding events, measured against the gold-standard data of the ST'09 development set. By sorting the events according to their confidence values, a tradeoff between precision and recall is obtained.

Using the confidence values for ranking, we have subsequently applied a cut-off threshold on the results, only keeping predictions with confidence values above the threshold. A systematic screening was performed between the interval of −1.7 and 1.3, using a step-size of 0.05 (60 evaluations). The results have been aggregated and summarized in Figure 2, depicting the average precision and recall values for each aggregated interval of 0.6 length. For example, a cut-off value between 0.10 and 0.70 (fourth interval) would result in an average precision rate of 70.0% and recall of 14.4%. Only taking the top ranked predictions, with a threshold above 0.7 (fifth interval), results in extremely high precision (91.9%) but only 4.8% recall. On the scale of EVEX, however, 4.8% recall would still translate to more than a million high-precision events.

### 3.3. EVEX Generalizations

As described in Section 2.1, the EVEX resource provides several algorithms to generalize gene symbols and their events, providing the opportunity to identify and aggregate equivalent events across various articles, accounting for lexical variants and synonymy. In a first step, a canonical form of the gene symbols is produced, increasing the proportion of symbols that can be matched to gene databases. This algorithm has previously been evaluated on the ST'09 training set, which specifically aims at identifying entities that are likely to match gene and protein symbol databases. By canonicalizing the symbols as predicted by BANNER, an increase of 11 percentage points in *F*-score was obtained [10].

The family-based generalizations have also been previously evaluated for both HomoloGene and Ensembl definitions. To expand the coverage of these generalizations, in this study, we have added definitions from Ensembl Genomes. The statistics on coverage of gene symbols, brought up to date by including the 2009–2011 abstracts, are depicted in Table 4. While only a small fraction of all unique canonical symbols matches the gene families from HomoloGene or Ensembl (Genomes) (between 3 and 6%), this small fraction accounts for more than half of all occurrences (between 51 and 61%). The family disambiguation algorithm thus discards a long tail of very infrequent canonical symbols. These findings are similar to the previous statistics presented by Van Landeghem et al. [10]. Additionally, the newly introduced families of Ensembl Genomes clearly provide a higher coverage: 8-9 percentage points higher than HomoloGene or Ensembl.

### 3.4. Event Refinement

By removing the chains of single-argument regulatory events, the refinement process simplifies and greatly reduces the heterogeneity in event structures, facilitating semantic interpretation and search for similar events. This process reduces the number of distinct event structures by more than 60%.

The main purpose of the event refinement algorithm, in combination with the pairwise view of the events, is to increase the coverage of finding related genes for a certain input query gene. When applying the algorithm as detailed in Section 2.3, the number of events with more than one gene symbol as direct argument increases from 1471 K to 1588 K, successfully generating more than a hundred thousand simplified events that can straightforwardly be parsed for pairwise relations.

It has to be noted, however, that the results of the refinement algorithm are merely used as an abstract layer to group similar events together and to offer quick access to relevant information. The original event structures as extracted by TEES are always presented to the user when detailed information is requested, allowing the user to reject or accept the inferences made by the refinement algorithm.

### 3.5. Biological Applications

The EVEX dataset and the associated web application have recently been applied in a focused study targeting the regulation of NADP(H) expression in *E. coli*, demonstrating the resource in a real-life biological use case, with encouraging results [20]. The Ensembl Genomes generalization was used to allow for homology-based inference, and the regulatory network extracted from EVEX was integrated with microarray coexpression data. As part of this study, 461 occurrences of two-argument events in the NADP(H) regulatory network were manually evaluated, with precision of 53%. This figure compares favorably with the BioNLP'09 Shared Task official evaluation results of 50% for binding events and 46% for regulation events, the only event types that allow more than one argument. The event occurrences that were judged to be correctly extracted were further evaluated for the correctness of the assignment of their arguments to Ensembl Genomes families: 72% of event occurrences had both of their arguments assigned to the correct family.

In a separate study, the suitability of the EVEX dataset and web application to the task of pathway curation was analyzed with a particular focus on recall [21]. When analysing three high-quality pathway models, TLR, mTOR and yeast cell cycle, 60% of all interactions could be retrieved from EVEX using the canonical generalization. A thorough manual evaluation further suggested that, surprisingly, the most common reason for a pathway interaction not being extracted is not a failure of the event extraction pipeline, but rather a lack of semantic coverage. In these cases, the interaction corresponds to an event type not defined in the ST'09 task and thus out of scope for the event extraction system. Only 11% of interactions in the evaluated pathways were not recovered due to a failure of the event extraction system. This result shows that the recall in EVEX, at least in the pathways under evaluation by Ohta et al., is clearly above the recall value published for the event extraction system in isolation. This increase can very likely be attributed to the volume of the event data in EVEX and the ability to aggregate several event occurrences into a single generalized event, where the failure to extract an individual event occurrence does not automatically mean the failure to extract the generalized event.

## 4. Web Application

To illustrate the functionality and features of the web application, we present a use case on a specific budding yeast gene, *Mec1*, which is conserved in *S. pombe*, *S. cerevisiae*, *K. lactis*, *E. gossypii*, *M. grisea,* and *N. crassa*. *Mec1* is required for meiosis and plays a critical role in the maintenance of genome stability. Furthermore, it is considered to be a homolog of the mammalian *ATR*/*ATM*, a signal transduction protein [22].

### 4.1. Gene Overview

 The main functionality of the EVEX resource is providing fast access to relevant information and related biomolecular entities of a gene or pair of genes of interest. (Analysis of large gene lists is currently not supported, as such a bioinformatics use case is already covered by the publicly available MySQL database.) The most straightforward way to achieve this is through the canonical generalization, searching for a gene symbol or a pair of genes separated by a comma.

**Figure 3:**
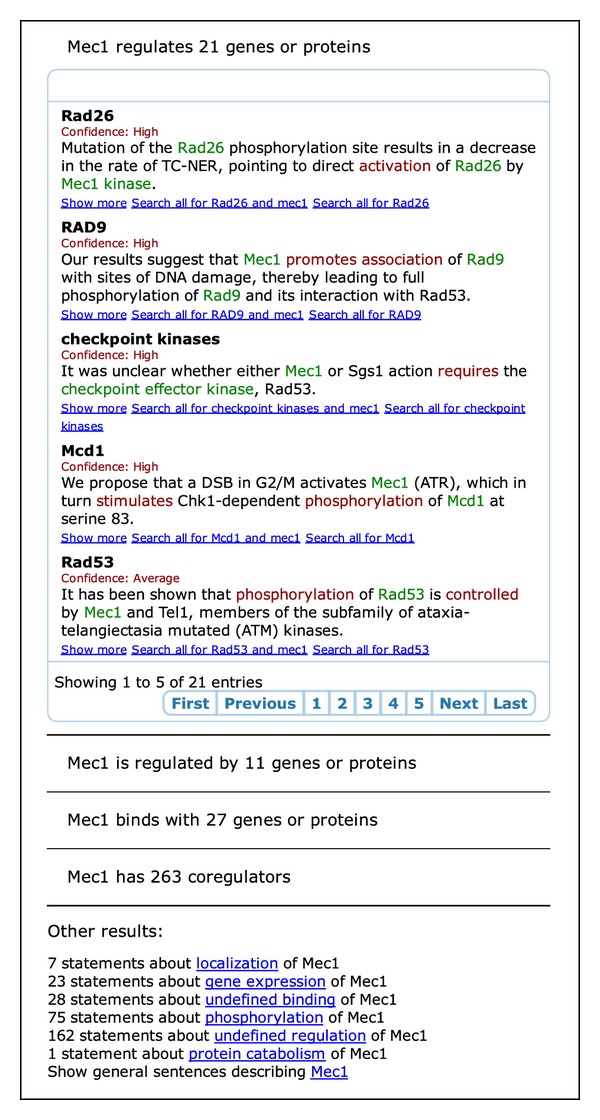
Search results for *Mec1* on the canonical generalization. An overview of directly associated genes is presented, grouped by event type. In the screenshot, only the box with regulation targets is shown, but the other event types may also be expanded. At the bottom, relevant links to additional sentences and articles are provided.

When typing the first characters of a gene symbol, a list of candidate matches is proposed, guiding the user to likely gene symbols found in text. The search page then automatically generates a listing of relevant biomolecular events, grouped by event type. At the top of the page, an overview of all regulators, regulated genes, and binding partners is provided, each accompanied with an example sentence. Further, coregulators are listed together with the number of coregulated genes (Section 2.5).  Figure 3 shows the results when searching for *Mec1*. This overview lists 21 regulation targets, 11 regulators, 27 binding partners, and 263 coregulators. Within each category, the events are ranked by confidence, ranging from (very) high to average and (very) low (Section 2.2). Further, example sentences are always chosen to be those associated with the highest confidence score.

**Figure 4:**
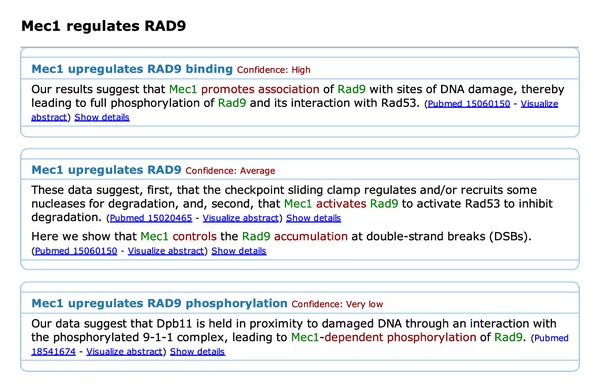
Detailed representation of all evidence supporting the regulation of RAD9 by Mec1. Regulatory mechanisms can have a certain polarity (positive/negative) and may involve physical events such as phosphorylation or protein-DNA binding.

Selecting the target *RAD9*, the web application visualises all event structures expressing regulation of *RAD9* by *Mec1* (Figure 4). This enables a quick overview of the mechanisms through which the regulation is established, which can have a certain polarity (positive/negative) and may involve physical events such as phosphorylation or protein-DNA binding. The different types of event structures are coarsely grouped into categories of similar events and presented from most to least reliable using the confidence scores.

**Figure 5:**
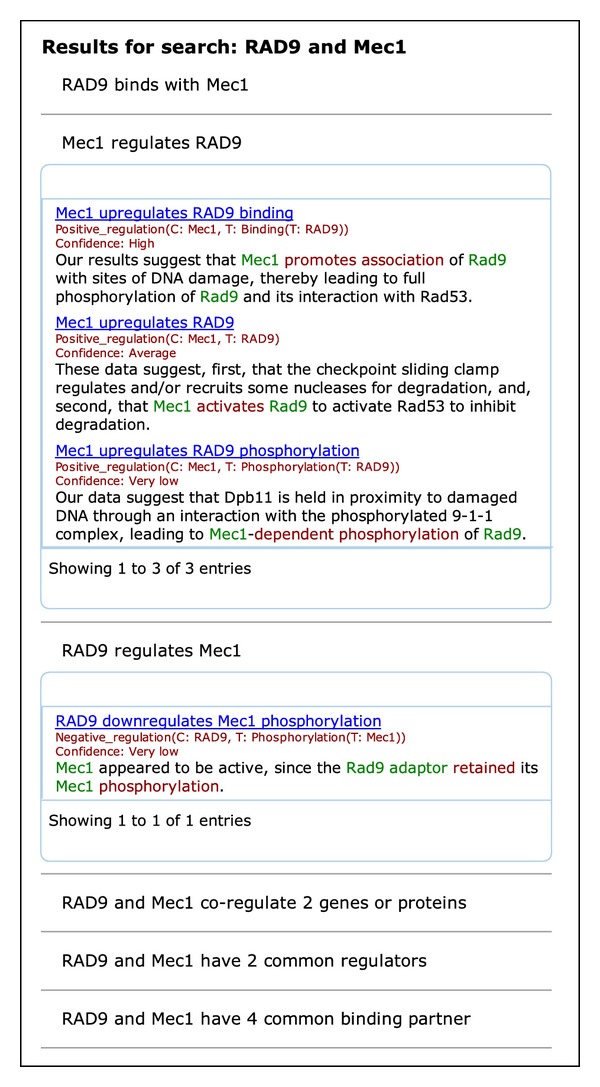
All events linking *Mec1* and *RAD9* through either direct or indirect associations. In the screenshot, only the regulation boxes are shown in detail, but the other event types may also be expanded. This page enables a quick overview of the mechanisms through which two genes interact, while at the same time highlighting false positive text mining results which can be identified by comparing confidence values and the evidence found in the sentences.

Exploring the relationship between *RAD9* and *Mec1* further, EVEX enables a search of all events linking these two genes through any direct or indirect association (Figure 5). This page provides conclusive evidence for a binding event between *RAD9* and *Mec1*. Further, both a *Mec1 regulates RAD9* and a *RAD9 regulates Mec1* event are presented. However, inspecting the sentences, the first one is obviously the only correct one. This illustrates the opportunity to use the large-scale event extraction results for pruning false positives of the text mining algorithm, as the false result only has 1 piece of evidence, and with a “very low” confidence, while the correct regulation is supported by 3 different evidence excerpts, two of which are of “high” confidence, and is thus displayed first.

Apart from the regulatory and binding mechanisms, the overview page also lists potential coregulations, enumerating targets that are regulated by both genes, such as *Rad53*. When accessing the details for this hypothesis, all evidence excerpts supporting both regulations are presented. Other indirect associations, such as common regulators and binding partners, can be retrieved equally fast.

Finally, the overview page of *Mec1* (Figure 3) contains additional relevant information including links to sentences stating events of *Mec1* without a second argument, grouped by event type. While these events incorporate only a single gene or protein and may not be very informative by themselves, they are highly relevant for information retrieval purposes, finding interesting sentences and articles describing specific processes such as protein catabolism or phosphorylation.

At the bottom of the overview page, a similar and even more general set of sentences can be found, providing pointers to relevant literature while still requiring manual analysis to determine the exact type of information. Such sentences, even though they contain no extracted events, may include useful background information on the gene such as relevant experimental studies, related diseases, or general functions and pathways.

### 4.2. Homology-Based Inference

 In comparative genomics, it is common practice to transfer functional annotations between related organisms for genes sharing sequence similarity [23, 24]. The EVEX resource provides such functionality for inferring interactions and other biomolecular events based on homology, by summarizing all events pertaining to a certain family when searching for one of its members (Section 2.1).

For example, instead of only looking at the information for one particular gene symbol as described previously, we can extend the search through Ensembl Genomes and retrieve information on homologous genes and their synonyms. The generated listings of regulators and binding partners are structured in exactly the same way as before, but this time each symbol refers to a whole gene family rather than just one gene name.

Conducting such a generalized search for *Mec1*, EVEX retrieves interaction information for *Mec1* and its homologs. The resulting page presents not only results for the symbol *Mec1*, but also for common symbols which are considered synonyms on the gene-family level, such as *ATR*. This type of synonym expansion goes well beyond a simple keyword query.

For each gene family present in the text mining data, a family profile lists all genes and synonyms for a specific family, linking to the authoritative resources such as Entrez Gene and the Taxonomy database at NCBI. While *ESR1* is a known but deprecated synonym of *Mec1* [25], it is not considered as a viable synonym of *Mec1*, considering *Esr1* generally refers to the family of estrogen receptors. The synonym disambiguation algorithm of Van Landeghem et al. [10], which is the basis of the gene family generalizations, will thus prevent *Esr1* from being used as a synonym for *Mec1*. Reliable synonyms found in text do however include *ATR* and *SCKL*.

The EVEX web application includes several distinct methods of defining gene families (Section 2.1), each accommodating for specific organisms and use cases. For example, Ensembl Genomes defines rather coarse grained families resulting in a family of 19 evolutionarily conserved genes, including the budding yeast gene *Mec1*, its mammalian *ATR* orthologs, and genes from green algae and Arabidopsis. In contrast, the corresponding family defined by HomoloGene only includes the 6 conserved *Mec1* genes in the Ascomycota.

### 4.3. Manual Inspection of Text Mining Results

An important aspect of the EVEX web application is the ability to retrieve the original sentences and articles for all claims extracted from literature. In the previous sections, we have described how EVEX can assist in the retrieval of directly and indirectly associated genes and proteins by generating summary overviews. However, to be applicable in real-life use cases and to be valuable to a domain expert, it is necessary to distinguish trustworthy predictions from unreliable hypotheses. For this reason, automatically generated confidence values are displayed for each extracted interaction, ranging from (very) high to average and (very) low. On top of those, the site always provides the opportunity to inspect the textual evidence in detail.

**Figure 6:**
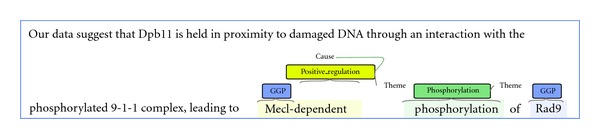
Visualization of a specific event occurrence by the stav text annotation visualiser. Genes and gene products (“GGPs”) are marked, as well as the trigger words that refer to specific event types. Finally, arrows denote the roles of each argument in the event (e.g. Theme or Cause). This visualization corresponds to the formal bracketed format of the event: *Positive-regulation(C: Mec1, T:Phosphorylation(T:RAD9))*.

Consider, for example, the phosphorylation of *RAD9*, regulated by *Mec1* (Figure 4). To allow a detailed inspection of this event, the web application integrates the *stav* visualiser [26], which was developed as a supporting resource for the ST'11 [2] (Figure 6). This open-source tool provides a detailed and easily graspable presentation of the event structures and the associated textual spans. To any user interested in the text mining details, this visualization provides valuable insights into the automated event extraction process. Additionally, the web application provides the opportunity to visualise whole PubMed abstracts with the *stav* visualiser, allowing a fast overview of event information contained within an abstract.

### 4.4. Site Navigation

 To easily trace back previously found results, a session-based search history at the righthand side of the screen provides links to the latest searches issued on the site. Further, a box with related searches suggests relevant queries related to the current page. Finally, the web application provides a powerful method to browse indirectly associated information, by allowing the retrieval of nested and parent interactions of a specific event. For example, when accessing the details of *Mec1*'s regulation of *RAD9* phosphorylation and selecting the phosphorylation event, evidence is shown for many parent events involving different regulation polarities and various genes causing this specific phosphorylation. As such, we quickly learn that *RAD9* phosphorylation has many different potential regulators, such as *Ad5*, *Ad12,* and *C-Abl*. This sort of explorative information retrieval and cross-article discovery is exactly the type of usage aimed at by the EVEX resource.

## 5. Conclusions and Future Work

This paper presents a publicly available web application providing access to over 21 million detailed events among more than 40 million identified gene/protein symbols in nearly 6 million PubMed titles and abstracts. This dataset is the result of processing the entire collection of PubMed titles and abstracts through a state-of-the-art event extraction system and is regularly updated as new citations are added to PubMed. The extracted events provide a detailed representation of the textual statements, allowing for recursively nested events and different event types ranging from phosphorylation to catabolism and regulation. The EVEX web application is the first publicly released resource that provides intuitive access to these detailed event-based text mining results.

As the application mainly targets manual explorative browsing for supporting research in the life sciences, several steps are taken to allow for efficient querying of the large-scale event dataset. First, events are assigned confidence scores and ranked according to their reliability. Further, the events are refined to unify different event structures that have a nearly identical interpretation. Additionally, the events are aggregated across articles, accounting for lexical variation and generalizing gene symbols with respect to their gene family. This aggregation allows for efficient access to relevant information across articles and species. Finally, the EVEX web application groups events with respect to the involvement of pairs of genes, providing the users with a familiar gene-centric point of view, without sacrificing the expressiveness of the events. This interpretation is extended also to combinations of events, identifying indirect associations such as common coregulators and common binding partners, as a form of literature-based hypothesis generation.

There are a number of future directions that can be followed in order to extend and further improve the EVEX web application. The core set of events can be expanded by also processing all full-text articles from the open-access section of PubMed Central. Further, as BioNLP methods keep evolving towards more detailed and accurate predictions, the dataset can be enriched with new information, for example, by including epigenetics data as recently introduced by the BioNLP'11 Shared Task [2, 27] and integrating noncausal entity relations [28, 29]. Additionally, gene normalization data can be incorporated, enabling queries using specific gene or protein identifiers [30]. Finally, a web service may be developed to allow programmatic access to the EVEX web application, allowing bulk queries and result export for further postprocessing in various bioinformatics applications.

## Figures and Tables

**Table 1:** Listing of the refinement rules, involving any nested combination of the three types of regulation: positive regulation (Pos), negative regulation (Neg) and unspecified regulation (Reg). Each parent event has a regulatory (T)heme argument and an optional (C)ause. The nested regulations are all regulations without a Cause and their detailed structure is omitted for brevity. In full, the first structure would read *Pos(C:geneA, T:Pos(T:geneB))* which is rewritten to *Pos(C:geneA, T:geneB)* with *geneA* and *geneB* being any two genes.

Original	Result	Example
*Pos(C, T:Pos)*	*Pos(C, T)*	BRs induce accumulation of BZR1 protein
*Pos(C, T:Reg)*	*Pos(C, T)*	PKS5 mediates PM H +- ATPase regulation
*Reg(C, T:Pos)*	*Pos(C, T)*	CaM regulates activation of HSFs
*Neg(C, T:Neg)*	*Pos(C, T)*	E2 prevented downregulation of p21

*Reg(C, T:Reg)*	*Reg(C, T)*	PDK1 is involved in the regulation of S6K

*Neg(C, T:Reg)*	*Neg(C, T)*	GW5074 prevents this effect on ENT1 mRNA
*Neg(C, T:Pos)*	*Neg(C, T)*	BIN2 negatively regulates BZR1 accumulation
*Reg(C, T:Neg)*	*Neg(C, T)*	The effect of hCG in downregulating ER beta
*Pos(C, T:Neg)*	*Neg(C, T)*	DtRE is required for repression of CAB2

**Table 2:** The most prevalent (refined) event patterns in the EVEX data, considering only events with more than one gene or protein symbol, and their recursively nested events. These aggregated patterns refer to any type of regulation (*Reg), to binding events between two genes (*Bind*), and to any physical event (*Phy*) concerning a single gene such as protein-DNA binding, protein catabolism, transcription, localization, phosphorylation, and gene expression. The first two columns refer to the percentage of event occurrences covered by the given pattern and the cumulative percentage of event occurrences up to and including the pattern. The right-most column depicts the extracted gene pair and a coarse classification of its association type. *A* and *B* refer to gene symbols, and bindings are represented with ×. Further, *A* > *B* means *A regulates B,* while *A* ≫ *B* expresses an indirect regulation.

Occ. [%]	Cum. occ. [%]	Event pattern	Gene pair
58.6	58.6	*Phy(T:A)*	—
15.0	73.6	*Reg(T:A)	—
8.4	82.0	*Reg(T:Phy(T:A))	—
8.0	90.0	*Bind(T:A, T:B)*	*A* × *B*
4.7	94.7	*Reg(C:A, T:B)	*A* > *B*
3.8	98.5	*Reg(C:A, T:Phy(T:B))	*A* > *B*
0.2	98.7	*Reg(C:*Reg(T:Phy(T:A)), T:Phy(T:B))	*A* ≫ *B*
0.2	98.9	*Reg(C:Phy(T:A), T:B)	*A* ≫ *B*
0.2	99.1	*Reg(C:Phy(T:A), T:Phy(T:B))	*A* ≫ *B*

**Table 3:** Indirect associations between gene *A* and gene *B*, established by combining binding and regulatory events through a common interaction partner gene *Z*. Bindings are represented with × and for regulations *A* > *B* means *A* regulates *B*.

Association	Interpretation
*A* > *Z* < *B*	*A* and *B* coregulate *Z*
*A* < *Z* > *B*	*A* and *B* are being regulated by *Z*
*A* × *Z* × *B*	*A* and *B* share a common binding partner *Z*

**Table 4:** Gene symbol coverage comparison, showing the number of distinct canonical symbols as well as the number of different occurrences covered, out of the total number of 40.3 M extracted gene symbols.

	Distinct symbols		Occurrences	
Canonical	1833.1 K	100.0%	40.3 M	100.0%
HomoloGene	68.2 K	3.7%	21.1 M	52.3%
Ensembl	60.0 K	3.2%	20.9 M	51.8%
Ensembl Genomes	100.6 K	5.5%	24.3 M	60.1%
